# Association of adolescent postural tachycardia syndrome classifications with anxiety: a cross sectional study

**DOI:** 10.1186/s13030-024-00301-z

**Published:** 2024-01-29

**Authors:** Midori Mizutani, Seiji Yoshida, Hidetaka Tanaka, Ginroku Yamawake, Atsuko Kubo, Yusuke Kurooka, Yoshitaka Ohta, Akira Ashida

**Affiliations:** 1 Department of Pediatrics, Hokusetsu General Hospital, Takatsuki, Japan; 2 Department of Pediatrics, Osaka Medical and Pharmaceutical University Hospital, 2-7, Daigakumachi, Takatsuki, Osaka 569-8686 Japan; 3 OD Hypotension Clinic Tanaka, Osaka, Japan; 4 Department of Pediatrics, Saiseikai Suita Hospital, Suita, Japan

**Keywords:** POTS, Anxiety, Autonomic Function, Adolescent, Frequency analysis

## Abstract

**Background:**

Postural tachycardia syndrome (POTS), a subset of orthostatic dysregulation, has been reported to be associated with anxiety. POTS can be classified into two forms based on the degree of tachycardia during orthostasis. Reportedly, POTS with decreased orthostatic heart rate increase is associated with suppressed cardiac parasympathetic activity and increased sympathetic activity in the supine position. In this study, the relationship between the two types of POTS and anxiety was evaluated in terms of autonomic function.

**Methods:**

Fifty-two patients (23 male, age 10–15 years) who were diagnosed with POTS at the Department of Pediatrics, Osaka Medical and Pharmaceutical University from 2019 to 2021, completed a standing test and were accordingly classified into a Su group, with tachycardia from the supine position and a low heart rate increase on standing, a SI group, with a high heart rate increase during standing. They then completed the State-Trait Anxiety Scale for Children (STAIC) questionnaire. Autonomic function was assessed by frequency analysis (MemCalc method) based on heart rate, blood pressure changes, heart rate and blood pressure variability during the orthostatic test.

**Results:**

Patients in the Su group had higher trait anxiety and state anxiety, lower cardiac parasympathetic activity (RR-HF) in the supine position, and greater variability in cardiac parasympathetic activity during orthostasis than were found for patients in the SI group. The Su group had a greater decrease in cardiac index on standing than that of the SI group.

**Conclusions:**

The Su group results may be partly attributed to chronically low venous return. We also found that patients in the Su group had low parasympathetic activity in the supine position, which may interact with the anxiety-prone characteristics of these patients. Therefore, it seems necessary to consider both physical and psychosomatic treatment approaches for patients with POTS.

## Background

Postural tachycardia syndrome (POTS) is one of the most frequent forms of orthostatic dysregulation (OD) in children. OD is a condition in which a person presents with symptoms of orthostatic ataxia—such as dizziness, headache and palpitations, when standing up from a supine or seated position—that is mainly caused by a malfunction of the autonomic regulatory function. Previous Japanese studies reported that the prevalence of OD increases before adolescence and peaks at 15% in adolescents aged 14–15 years [1].

POTS is defined by the Japanese Society of Psychosomatic Pediatrics (JSPP) as an increase in heart rate ≥ 35 beats/min or heart rate during standing ≥ 115 beats/min without orthostatic hypotension [2]. The false-positive rate of the standing test using these criteria was 4.3% in healthy Japanese children (6/140 subjects aged 6–18 years) [3]. These criteria are very close to those proposed by the American Autonomic Society (increase in heart rate ≥ 30 beats/ min or heart rate during standing ≥ 120 beats/min) [4].

POTS has long been diagnosed as a psychological problem, such as chronic anxiety, due to symptoms such as fainting, fatigue, palpitations, and anxiety [5]. Even after POTS became more widely recognized in recent years, there have been several reports of POTS patients have more anxiety than general subjects [6, 7]. Therefore, we aimed to clarify the association between POTS and anxiety in greater detail.

Reports have classified POTS into two groups: standing-induced tachycardia (SI group; increase in HR ≥ 35 beats/min) and supine tachycardia (Su group; standing HR ≥ 115 beats/min with standing-induced HR increase < 35 beats/min) [8]. A Su group with tachycardia in the supine position was reported to have decreased parasympathetic function and increased sympathetic function in the supine position. We hypothesized that the Su group would be characterized by a tendency toward anxiety because anxiety and tachycardia are likely to be associated. Therefore, in this study, we evaluated autonomic function and an anxiety scale in the Su and SI groups and assessed their relationships with anxiety.

## Methods

### Participants

We investigated children with orthostatic symptoms referred to Osaka Medical and Pharmaceutical University Hospital for further examination during 2019–2021. According to the JSPP clinical guideline criteria for POTS, 52 children (23 male and 29 female), age 10–15 years (mean ages, 13.0 ± 1.0 years and 13.4 ± 1.2 years, respectively), met the criteria. All patients underwent general physical examination, including neurological examination, 12-lead electrocardiogram (ECG), and blood tests including hematologic analysis, serum electrolytes, and serum thyroxine. No abnormalities, including anemia or febrile illness, were found in any of the patients. No patient had anxiety disorders or other psychiatric disorders, and none had intellectual disabilities that prevented them from answering the questionnaire. In addition, to investigate whether there was a tendency toward neurodevelopmental disorders (autism spectrum disorder: ASD), the patient’s parent was asked to complete the Autism-Spectrum Quotient (AQ), a test in which a score of 25 or higher is diagnostic of ASD [9, 10]. One response was omitted in each group, but the percentage of ASD was not significantly different (Table 1). School attendance was evaluated in three groups: less than 1 day per week, 2 or 3 days per week, and 4 or more days per week. There were 11, 1, and 3 patients in the Su group and 12, 7, and 18 patients in the SI group, respectively, with significant differences (*p* = 0.03).

**Table 1:** Subject characteristics

		Su group (mean ± SE)	SI group (mean ± SE)	*P*
M/F		2/13	*21/16*	0.004*
Age (years)		13.2 ± 0.2 (11–15)	13.3 ± 0.3 (10–15)	0.840
ASD (AQ ≥ 25) (%)		14.3 (2/14)	30.6 (11/36)	0.303
Height (cm)		156.2 ± 1.3	160.4 ± 1.4	0.082
Body weight (kg)		43.6 ± 1.3	49.4 ± 1.7	0.051
BMI-SD		-0.8 ± 0.2	-0.3 ± 0.2	0.173
HR (beats/min)	Supine	91.7 ± 1.6	72.2 ± 1.5	< 0.001*
	Standing	119.9 ± 1.8	114.2 ± 1.4	0.164
ΔHR (beats/min)		28.1 ± 1.0	42.1 ± 0.9	< 0.001*
SBP (mmHg)	Supine	114.5 ± 1.8	109.4 ± 1.6	0.073
	Standing	114.1 ± 2.5	112.0 ± 1.8	0.500
DBP (mmHg)	Supine	67.7 ± 1.0	63.7 ± 1.0	0.023*
	Standing	75.9 ± 1.7	76.2 ± 1.8	0.837
RR-HF (mSec^2^)	Supine	356.7 ± 84.9	669.6 ± 71.4	0.010*
	Standing	96.5 ± 24.4	95.9 ± 13.0	0.785
RR-LF/HF	Supine	1.5 ± 0.2	1.3 ± 0.1	0.391
	Standing	3.4 ± 0.5	4.6 ± 0.6	0.369
DBP-LF (mmHg^2^)	Supine	6.6 ± 1.1	6.8 ± 0.6	0.832
	Standing	15.8 ± 2.2	11.9 ± 0.9	0.143
Pulse pressure (mmHg)	Supine	46.9 ± 1.5	45.7 ± 1.3	0.792
	Standing	38.3 ± 2.1	35.8 ± 1.9	0.935
SV (ml)	supine	48.3 ± 4.8	59.9 ± 3.6	0.027*

*M* Male, *F* Female, *ASD* Autism spectrum disorder, *BMI* Body mass index, *HR* Heart rate, Δ*HR* Change in HR from supine to standing, *SBP* Systolic blood pressure, *DBP* Diastolic blood pressure, *RR-HF* High-frequency component of RR interval variability, *RR-LF/HF* Ratio of the LF to HF components of RR interval variability, *DBP-LF* Low-frequency component of DBP variability, *SV* Stroke volume, *Su* Supine tachycardia, *SI* Standing-induced tachycardia

Values are expressed as mean ± SE. *, *p* < 0.05 between Su and SI groups

### Study design

All participants completed a standing test prior to the start of drug therapy and were accordingly classified into the two subsets of POTS. Autonomic function was assessed by frequency analysis (MemCalc method) based on heart rate, blood pressure changes, heart rate, and blood pressure variability during the orthostatic test. They then completed the State-Trait Anxiety Scale for Children (STAIC) questionnaire. Autonomic function and STAIC results were compared between two subsets of POTS.

Informed consent was obtained from all participants and parents. The study was approved by the Ethics Committee of Osaka Medical and Pharmaceutical University (approval no. 2662–2).

### Autonomic assessment

All patients performed an active standing test as previously reported [11]. Briefly, patients were quietly seated for 15 min in a waiting room before the actual measurement started. The test was performed in the morning in a soundproof room at temperatures between 23 °C and 25 °C. A Finometer cuff (model 1; FMS, Amsterdam, Netherlands) was placed on the middle phalanx of the third finger of the right hand. To ensure optimal Finometer blood pressure (BP) measurement, we used appropriate cuff sizes (S or M), according to the manufacturer’s instructions.

Before the test, patients were instructed to stand up quickly and independently, without using their right hand, such as not to affect the Finometer recording. After 10 min in the supine position, the patients were asked to stand up actively by themselves and remain standing for 7 min. The diagnostic criterion for POTS was evaluated by heart rate after 3 min of standing. Originally, the guideline required standing for 10 min, but in consideration of physical strain, standing was limited to 7 min in this study. All patients stood within 3–4 s. During the active standing test, patients were monitored using continuous non-invasive finger arterial pressure measurement (Finometer).

Finger arterial BP and ECG signals were digitally stored on a personal computer system (Dynabook; Toshiba, Japan) during the entire test period. The default signal analysis program (Beat Scope; FMS, Amsterdam, Netherlands) provided data on sequential R-R intervals of the ECG (ms), beat-to-beat systolic and diastolic blood pressure (SBP and DBP; mmHg), cardiac output (CO), and stroke volume (SV). Sequential RR intervals, SBP, and DBP were obtained in the supine position (4 min) and late period of standing (4–7 min after standing). SBP and DBP values were averaged over 4 min in the supine position and 4 to 7 min after standing.

We evaluated the variability of the RR interval and BP of high frequency (HF; 0.15–0.4 Hz) and low frequency (LF; 0.04–0.15 Hz) components as an index of cardiovascular autonomic function. Generally, it is accepted that the HF component of RR interval variability (RR-HF) is mediated by cardiac parasympathetic tone generated by respiration, whereas the LF component is mediated by both cardiac sympathetic and parasympathetic tones. The ratio of the LF to HF components of RR interval variability (RR-LF/HF) is considered an index of cardiac sympathetic tone [12–15]. Contrastingly, the HF component of BP variability has been regarded as a mechanical consequence of respiration [12] and the LF component of BP variability has been reported to parallel sympathetic vasomotor activity [12, 16]. Regarding the influence of stroke volume, DBP variability can be evaluated more accurately using sympathetic vasomotor activity than SBP variability. Therefore, we used the LF component of DBP variability (DBP-LF) to evaluate sympathetic vasomotor activity. Spectral analysis using the maximum entropy method [17] (MemCalc for Windows version 1.2, Suwa Trust, Tokyo, Japan) was applied to the time-series data for each variable.

The Finometer includes the Modelflow method to derive continuous CO from finger pressure [18].

We calculated the cardiac index (CI) in each subject by the following equation:

$${\text{CI}}\left({\text{L}}\right)/{\text{min}}/{{\text{m}}}^{2}={\text{CO}}\left({\text{L}}/{\text{min}}\right)/\mathrm{body surface area }\left({{\text{m}}}^{2}\right)$$

$$\mathrm{Bodysurfacearea}\left(\text{m}^2\right)=\left.\left(\text{bodyweight}\left(k\text{g}\right)\times\text{height}\left(\text{cm}\right)/3600\right)^{0.5}\right)$$

[19].

### Questionnaire

We used a self-administered questionnaire, The State-Trait Anxiety Inventory for Children (STAIC) [20]. The STAIC was developed based on the State-Trait Anxiety Inventory (STAI) for adults. Later, in 1983, Soga standardized the STAIC in Japanese [21]. It can measure both state (temporary anxiety in the moment) and trait (anxiety as a personality trait, that is, a relatively stable individual's tendency to react) anxiety. We administered the STAIC test to the participants before the active standing test.

### Data analysis

We divided the patients into two groups: a standing-induced tachycardia (SI) group, defined as an increase in heart rate during standing of ≥ 35 beats/min, and a supine tachycardia (Su) group, defined as an increase in heart rate during standing of < 35 beats/min with a standing heart rate of ≥ 115 beats/min.

We statistically evaluated the differences in the SI and Su groups using autonomic function and anxiety scale data.

Statistical analysis was performed using JMP pro14 (SAS Institute Inc., NC, USA). Data are presented as mean ± SE unless otherwise noted. Comparisons were made between the two POTS groups using the Student’s t-test and Fisher’s exact test. Statistical significance was set at *p* < 0.05.

## Results

This study included 52 children (23 male and 29 female), age 10–15 years (mean ages 13.0 ± 1.0 years and 13.4 ± 1.2 years, respectively). Fifteen patients were classified in the Su group and 37 in the SI group. In terms of sex, significantly more were female in the Su than the SI group. There were no significant differences between the two groups in terms of height, weight, or BMI-SD. In the supine position, SV was significantly lower in the Su than in the SI group, and DBP was significantly higher in the Su than in the SI group (Table 1). Physical symptoms during the standing test were not significantly different between the two groups. Specifically, they were dizziness (Su group 67%, SI group 81%), headache (Su group 53%, SI group 35%), palpitations (Su group 53%, SI group 51%), and fatigue (Su group 89%, SI group 100%).

**Fig. 1:**
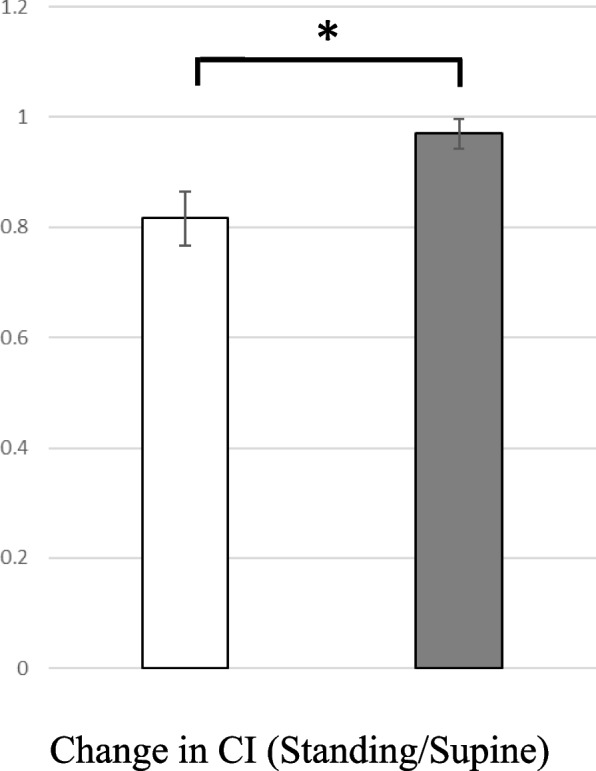
Change in cardiac index upon standing in the Su group (□) and the SI group (■). The vertical line indicates standing/supine ratio. Error bar, mean ± SE. ^*^ *P* < 0.05

When upright, the reduction in the CI was significantly greater in the Su than the SI group (0.82 ± 0.05 vs 0.97 ± 0.03, respectively, *p* < 0.05) (Fig. 1).

**Fig. 2:**
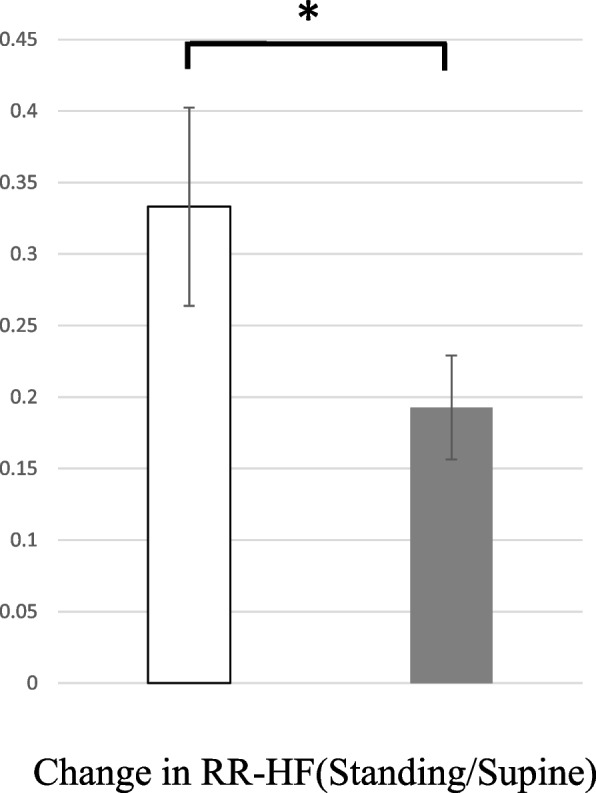
RR-HF (standing/supine) in the Su (□) and SI groups (■). Error bar, mean ± SE. ^*^ *P* < 0.05

For heart rate variability, in the supine position, the RR-HF was significantly lower in the Su than in the SI group (Table 1). The change in RR-HF during standing was also greater in the Su than in the SI group (0.33 ± 0.07 vs 0.19 ± 0.04, respectively, *p* < 0.05) (Fig. 2).

For BP variability, DBP-LF in the supine and standing positions did not differ between the two groups. The pulse pressures in the supine and standing positions did not differ between the two groups.

**Fig. 3:**
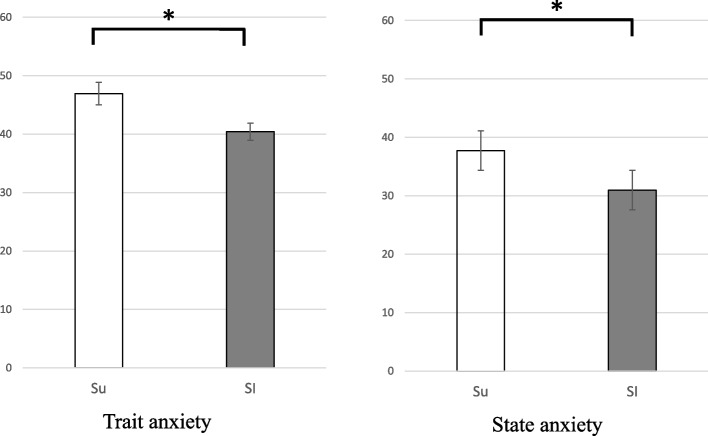
Trait anxiety and state anxiety in the Su (□) and SI groups (■). Error bar, mean ± SE. ^*^ *P* < 0.05

Both trait anxiety and state anxiety were higher in the Su group than in the SI group (46.93 ± 1.93 vs 40.45 ± 1.49, 37.73 ± 2.50 vs 30.97 ± 1.27, respectively, *p* < 0.05) (Fig. 3).

## Discussion

POTS is characterized by a marked increase in heart rate during standing without apparent hypotension. POTS was first reported as a sympathetic tonic response [22]. Subsequently, this response to orthostasis has been increasingly reported and studied in many countries in recent years. The excessive increase in heart rate in POTS is attributed to the low baroreflex system, which is activated by reduced venous return to the heart [23].

Yoshida classified POTS into SI and Su groups and referred to their hemodynamics [8]. Both the SI and Su groups had lower RR-HF in the supine position, reflecting the parasympathetic function of the heart, compared to that of controls, indicating the presence of reduced vagal tone via the low-pressure baroreflex. However, they also reported different hemodynamic characteristics in both groups.

This means that the Su group, which tended to be more tachycardic in the supine position, had a lower RR-HF in the supine position than did of the SI group and tended to have a greater change in RR-HF when standing. Similar results were observed in this study, indicating that children in the Su group had more suppressed vagal activity and responded more significantly to the stress load of standing. Yoshida reported reduced central blood volume as a factor in the inactivation of the supine low baroreflex system in the Su group [8]. The “low-flow” POTS proposed by Stewart [24] is also characterized by a decrease in central blood volume, similar to the hemodynamic characteristics of the Su group. In this study, the Su group also showed a lower rate of change in CI than was seen in the SI group. This likely reflects a lower venous return to the heart when standing. Furthermore, even though there was no difference in body size between the Su and SI groups, SV was significantly lower in the Su group. There are three variables that affect SV, contractility, preload, and afterload [25]. The reason for the lower SV in the Su group could be attributed to a lower preload due to the hypovolemic state. Therefore, we considered it necessary to increase central blood volume in the Su group.

In this study, we considered two hypotheses regarding the pathogenesis in the Su group. One hypothesis is that higher anxiety may lead to lower resting parasympathetic function, resulting in the pattern of tachycardia tendency in the supine position in the Su group. The results of this study showed that the Su group had higher anxiety scale and lower cardiac parasympathetic activity in the supine position than did the SI group. A report on the relationship between high-anxiety personality and autonomic function, evaluating autonomic function from heart rate variability, found that parasympathetic function was reduced in patients with anxiety disorders [26]. Our findings are consistent with the these results.

Another hypothesis is that physical state may influence psychological anxiety. There are several reports of anxiety among POTS patients, indicating that anxiety in patients with POTS is caused by biological rather than psychological factors [27]. Patients with POTS have increased vigilance and anxiety related to their perception of cardiac symptoms [28]. The results of this study suggest that the Su group has a lower central blood volume than the SI group, thus the patients are constantly tachycardic and tachycardia is more likely to be enhanced during daily activities. POTS patients have increased vigilance and anxiety to their perception of cardiac symptoms, suggesting that the Su group is more likely to experience high anxiety.

In both hypotheses, it seems anxiety is higher in POTS patients, and studies focusing on psychological anxiety symptoms suggest that patients with POTS are more anxious than normal patients [29]. Although the anxiety symptoms themselves may be a strong physical alertness unique to POTS patients, it is clinically important to consider the reported comorbidity of anxiety disorders in patients with POTS [7].

Our results indicate that, clinically, patients in the Su group must be aware of high levels of anxiety and decreased central blood volume. Patients in the Su group attended significantly fewer days of school, suggesting that they experience disruption to their daily lives.

The study has several limitations. This study has an institutional bias in that it included patients who visited a single university hospital. Regarding the assessment of autonomic function, adrenergic receptor sensitivity may be an important factor that is reportedly influenced by age [30]. Because the age of the participants in this study was similar between the two groups, we considered adrenoceptor sensitivity differences to be negligibly small.

Another factor is the evaluation of the central blood volume, which we argue is important for supporting this hypothesis. However, accurate measurements require invasive methods, such as dye dilution. In this study, we did not measure the central blood volume of our pediatric patients with POTS. Instead, CI, an indicator of central blood volume, was used. The CI data were automatically detected by the built-in software of the Finometer (Beat Port™), but its reliability with respect to absolute values in children is unknown. Therefore, only the CI ratio between the standing and supine positions was calculated and evaluated.

There was also a difference in the sex ratio between the Su and SI groups, with the Su group having a higher proportion female. In general, girls report higher levels of anxiety symptoms than boys [31], and this difference is a limitation of this study.

Another limitation of this study is the inability to discriminate whether the palpitations in the STAIC questionnaire were due to POTS symptoms or anxiety. Another limitation is its inability to assess the presence or absence of secondary sexual characteristics that might affect autonomic nervous function.

## Conclusion

In this study, two groups of patients with POTS were examined in terms of autonomic nervous system function and psychological aspects. The results showed that the Su group, which had higher tachycardia from the supine position, had lower resting cardiac parasympathetic function and higher levels of anxiety. These results indicate that anxiety in the Su group may have a biological basis, and treatment should address both the psychological and physical aspects.

The association between anxiety and the autonomic nervous system in patients with POTS remains controversial. In this study, we were able to provide evidence for this association, which may provide clinical implications for treatment strategies for POTS.

## Data Availability

The anonymized data underlying this article will be shared upon reasonable request to the corresponding author.

## Abbreviations

POTS: Postural tachycardia syndrome
OD: Orthostatic dysregulation
HR: Heart rate
SBP: Systolic blood pressure
DBP: Diastolic blood pressure
LF: Low frequency
HF: High frequency
CO: Cardiac output
CI: Cardiac index
SV: Stroke Volume
ASD: Autism spectrum disorder
BMI: Body mass index
